# Ecotypic differentiation matters for latitudinal variation in energy metabolism and flight performance in a butterfly under climate change

**DOI:** 10.1038/srep36941

**Published:** 2016-11-15

**Authors:** Hans Van Dyck, Marie-Jeanne Holveck

**Affiliations:** 1 Earth and Life Institute, Behavioural Ecology & Conservation Group, Université catholique de Louvain (UCL), Louvain-la-Neuve, Belgium

## Abstract

Life histories of organisms may vary with latitude as they experience different thermal constraints and challenges. This geographic, intraspecific variation could be of significance for range dynamics under climate change beyond edge-core comparisons. In this study, we did a reciprocal transplant experiment between the temperature-regimes of two latitudes with an ectotherm insect, examining the effects on energy metabolism and flight performance. *Pararge aegeria* expanded its ecological niche from cool woodland (ancestral) to warmer habitat in agricultural landscape (novel ecotype). Northern males had higher standard metabolic rates than southern males, but in females these rates depended on their ecotype. Southern males flew for longer than northern ones. In females, body mass-corrected flight performance depended on latitude and thermal treatment during larval development and in case of the southern females, their interaction. Our experimental study provides evidence for the role of ecological differentiation at the core of the range to modulate ecophysiology and flight performance at different latitudes, which in turn may affect the climatic responsiveness of the species.

Many species respond to current climate change by range shifts. Such effects are most easily observed at the expanding range edge^1^. Newly colonized populations at the range edge may differ in phenotypic traits from long-established populations at the core of the range^2^. Dispersive individuals that are more likely to colonize new sites can be a non-random sample of the population^3^. There can be further selection on functional traits in new populations at the moving edge creating stronger differentiation between edge and core populations^2^. Studies comparing phenotypic traits between edge and core populations have been helpful to better understand range limits and expansion under climate change^2^,^4^, but changing climatic conditions may also change ecological and evolutionary responses across an organism’s range. Therefore, distributions of phenotypic variation may also change at the core of the range and not exclusively at the expanding edge under climate change.

Several mechanisms contribute to geographic changes in phenotypic variation under climate change including phenotypic plasticity, mutations and evolution, evolution of increased phenotypic plasticity or invasion of southern phenotypes/genotypes^5^,^6^. The altered phenotypic composition may, in turn, influence responses at the population level and range dynamics at the species level. There is growing interest for studying phenotypic and genetic clines within the context of ecological and evolutionary responses to climate change^7^.

At different latitudes, organisms are faced with different climatic conditions favoring specific life-history traits (or trait values). Such climate-related patterns are of particular significance for ectotherms^8^. Along latitudinal clines, populations of ectotherms are faced with different activity time budgets and different trade-offs between current and future reproduction. As predicted by the metabolic cold adaptation hypothesis, metabolic rates of ectotherms (including many insects) from cold environments tend to be higher compared to those from warm environments^9^. Usually, these patterns are related to selection on growth rates and development time. However, recent work on *Drosophila* showed an alternative explanation for the same pattern; selection for survival in highly thermally variable habitats could also drive the evolution of respiratory metabolic rate in a pattern that is consistent with metabolic cold adaptation, but independent of selection on growth rate^10^. Independent of juvenile or larval growth, such variable thermal conditions may be highly relevant for adult activity and locomotor performance in individuals of ectothermic species at different latitudes, or generally in different thermal environments.

Here we are interested in biogeographic, intraspecific variation in energetic constraints and management in adult insect ectotherms^11^, and more particularly in flying heliotherms like butterflies. Their body temperature is strongly influenced by solar radiation, ambient temperature and wind speed through behavioural thermoregulation; body temperature will equal ambient temperature only in the absence of direct solar radiation. In temperate regions, values for the voluntary flight activity range lie well above the average ambient temperature^12^. Most adult activities require active flight, but insect flight is metabolically costly^13^. Average ambient temperature and solar radiation decrease with increasing latitude reducing the thermal window for butterfly activity. Moreover, weather conditions become more variable with increasing latitude, affecting potentially plastic responses and their evolution for energetic needs and physiological performance^14^. Weather and its variability influence the occurrence of flight and inactivity and the duration of flight bouts in butterflies^15^. Reestablishing metabolic activity to resume adult activity and coordinated movement is energetically intensive requiring significant quantities of ATP to restore homeostasis^10^. Hence, reduced activity budgets under more variable weather conditions at higher latitude can be compensated by an increased metabolic rate under conditions that allow adult activity.

Independent of latitudinal variation, different habitat types can also be associated with significant intraspecific variation in energetics and locomotor performance. Therefore, species with ecotypes^16^ differing in thermal physiology and mobility that occur at the same latitude are of particular interest in the context of climate change. Here we addressed the issue of habitat-related energy metabolism and flight performance at two different latitudes within the core range of the Speckled wood (*Pararge aegeria* L.). The range of this butterfly has expanded northward^17^, but in NW-Europe it recently also filled its range by niche expansion from woodland to agricultural landscape^18^. Common garden experiments and reciprocal transplant experiments have pointed to several adaptive differences between populations of woodland and agricultural landscape origin^19^,^20^. Woodlands are more humid, cooler and thermally buffered environments, whereas *P. aegeria* habitat in agricultural landscape is drier, warmer, but also more variable, and much more fragmented^19^,^21^.

In the laboratory, we simulated a reciprocal transplant experiment between two latitudes by rearing offspring of females of both ecotypes (woodland and agricultural populations) at the average temperature of the northern latitude (Belgium) and of the southern latitude (France) according to a full factorial design. There is latitudinal variation in morphology (e.g., larger adults with latitude), but the steepness of the gradients varies between the ecotypes (e.g., stronger increase in size in agricultural compared to woodland ecotype^22^). We measured standard metabolic rate (SMR) and quantified flight performance (i.e., flight endurance, measured as distance flown under stimulation) and simultaneously flight metabolic rate (FMR) in a flight mill in order to test for sex-specific latitude and ecotype effects after larval development under northern or southern thermal conditions.

Because of the short adult lifespan in *P. aegeria*^23^, weather-related interruptions of flight activity are assumed to be of considerable biological significance. Latitude-related constraints in adult activity budget and in higher synchronization of adult emergence in the north^24^ lead to the prediction of increased metabolic rates in northern compared to southern populations. This effect is predicted to be stronger in males because *P. aegeria* is a monandrous species with an increasing degree of protandry in the north^25^. Although male behaviour differs between woodland and agricultural populations, there is no evidence of differences in SMR between males adopting different mate-locating behaviours^26^. Similar to males, northern females are predicted to have an increased metabolic rate than southern females. However, taking into account ecotype-related thermal differences in females^19^, metabolic rates could be more canalized in females of woodland origin (i.e., more buffered thermal environment) compared to females of agricultural landscape origin. We predict higher plasticity in metabolic rates in individuals of agricultural landscape origin – which may interfere with latitudinal variation – as thermal conditions in *P. aegeria* habitat in agricultural landscape is more variable compared to cooler and thermally buffered habitat in woodlands^21^.

Females are the dispersive sex in *P. aegeria* responsible for between-population exchange and colonization^27^. Female flight performance can be traded-off against life-history traits like fecundity^17^ and longevity (but in an ecotype-specific way^20^). Males and females differ in flight pattern corresponding to different flight performances and energetic needs^27^. In another butterfly, the relationship between FMR and flight duration was indeed positive in females, but not in males^28^. So, northern females are predicted to have stronger performances for flight endurance and corresponding FMR compared to southern ones as they have to deal with a reduced time budget to spread their eggs across suitable host plants. Hence, northern females should be more mobile compared to southern conspecifics^29^, but under the assumption of local adaptation, mobility may be compromised when reared under cooler conditions for southern individuals and warmer conditions for northern individuals. However, predictions are different for males. At low ambient temperatures, males principally adopt a territorial perching strategy, while they adopt a patrolling, searching strategy with longer periods of flight when ambient temperature is highest^30^. Both behavioural strategies require different flight patterns^31^; flight endurance, as measured here in a flight mill, better reflect flight performance during patrolling rather than for territorial perching, which principally relies on acceleration and maneuverability^27^. Assuming higher specialization on perching in the north and in cooler woodland habitat compared to warmer agricultural habitat, we consequently predict lower flight endurance in the north, and particularly so in the woodland ecotype.

## Results

Tables 1 and 2 summarize the statistical analyses of SMR, flight performance and FMR (for all three we controlled for body mass, held as covariable in the models) relative to latitude, ecotype and developmental treatment for males and females, respectively.

### Standard metabolic rate

**Figure 1:**
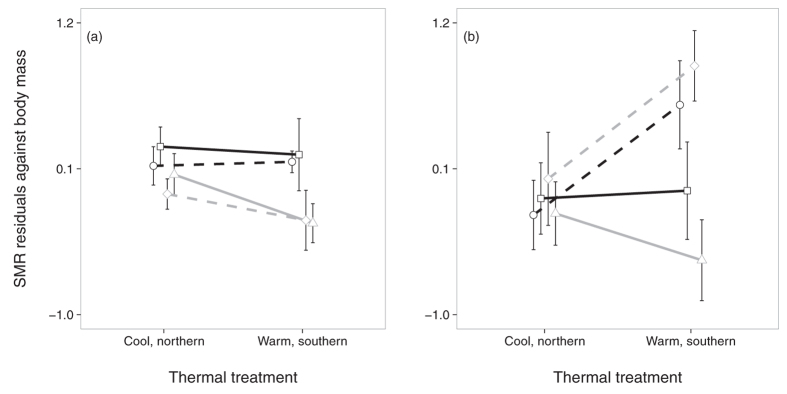
Standard metabolic rate SMR (residuals against body mass) of males (**a**) and females (**b**) by thermal treatment, latitude and ecotype of origin (north-woodland: squares, plain black line; north-agri: circles, dashed black line; south-woodland: triangles, plain grey line; south-agri: diamonds, dashed grey line). Means ± 1 SE.

As predicted, the results for SMR were very different in adult males and females (Fig. 1a,b). Northern males had higher SMR than southern males, independent of habitat and developmental temperature. In females, there was no latitudinal signal, but a significant interaction effect of habitat by developmental temperature. Independent of latitude, SMR increased strongly with developmental temperature in individuals of agricultural landscape origin only; females of woodland population origin did not show a significant response to developmental temperature. In females – but not in males – there was also a significant positive effect of age at testing and of the ambient temperature at testing.

### Flight performance

**Figure 2:**
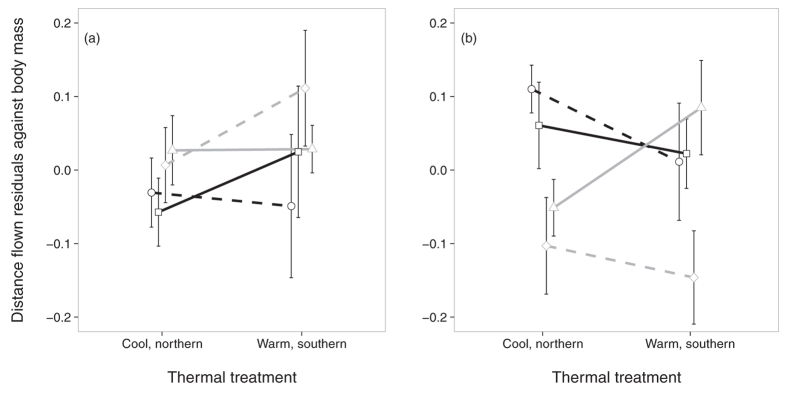
Distance flown (residuals against body mass) by males (**a**) and females (**b**) by thermal treatment, latitude and ecotype of origin (north-woodland: squares, plain black line; north-agri: circles, dashed black line; south-woodland: triangles, plain grey line; south-agri: diamonds, dashed grey line). Means ± 1 SE.

As predicted, flight distance patterns differed between the sexes (Fig. 2a,b). While the flight performance of males depended on latitude only, the flight performance of females was much more sensitive to the combined effects of latitude, habitat type and developmental temperature. Northern males flew shorter distances than southern males. In contrast, northern females flew longer distances after development under cool, northern developmental temperature, and *vice versa* for southern females, but the latter was only significant in females of woodland origin as there was no significant difference relative to developmental temperature in southern females of agricultural landscape origin. In both sexes, the distance flown increased with flight duration.

### Flight metabolic rate

**Figure 3:**
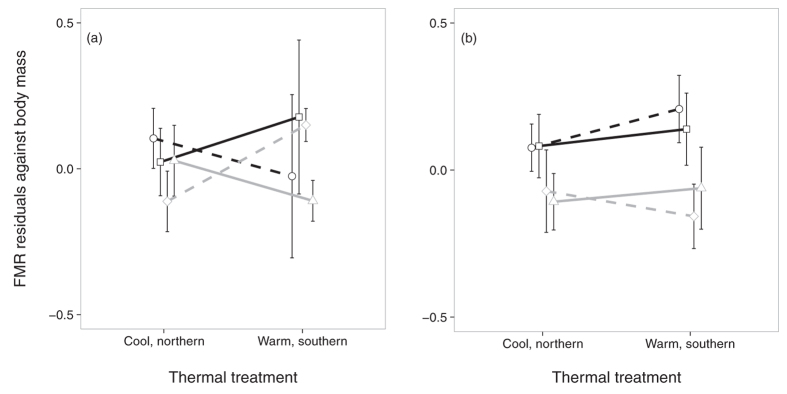
Flight metabolic rate FMR (residuals against body mass) of males (**a**) and females (**b**) by thermal treatment, latitude and ecotype of origin (north-woodland: squares, plain black line; north-agri: circles, dashed black line; south-woodland: triangles, plain grey line; south-agri: diamonds, dashed grey line). Means ± 1 SE.

Again, sexes differed in their FMR patterns. Northern females tended to have higher FMR, independent of habitat and developmental temperature (Fig. 3b). The results for males (Fig. 3a) turned out to be more complicated with habitat specific effects but in interaction with the thermal treatment. Under southern thermal conditions FMR was higher in southern males of agricultural ecotypes compared to woodland ecotypes and in northern males FMR tended to be actually higher in the woodland ecotype compared to the agricultural ecotype. However, this difference was only true for males of southern origin and disappeared at northern thermal conditions (Fig. 3a) explaining a tendency for a three-way interaction (Table 1). In males – and not in females (Table 2) – FMR also increased with flight duration and decreased with age.

### Relationship between SMR and FMR

FMR was 6.5-fold and 5.5-fold higher than SMR for males and females, respectively (mean ± 1 SD; FMR males (N = 95 males): 1.10 ± 0.50 ml CO_2_h^−1^; FMR females (N = 111): 1.11 ± 0.47 ml CO_2_h^−1^ – SMR males (N = 151): 0.17 ± 0.08 ml CO_2_h^−1^; SMR females (N = 156): 0.20 ± 0.09 ml CO_2_h^−1^). In both sexes, the models for the difference between FMR and SMR gave qualitatively similar results than the models of FMR (See Supplementary information).

SMR correlated negatively with distance flown in males (Pearson *r*_91_ = −0.27, *P* < 0.01), but not in females (*r*_108_ = −0.12). FMR correlated positively with distance flown in both sexes (males: *r*_93_ = 0.61, females: *r*_109_ = 0.57, both *P* < 0.001). SMR and FMR did not correlate with each other in either sex (males: *r*_93_ = −0.06; females: *r*_108_ = 0.09). These two-by-two correlations among SMR, FMR and distance flown did not differ across different latitudes and habitats (See Supplementary information). In males, the decrease of flight distance with SMR was similar at both latitudes (See Supplementary information).

## Discussion

Here we focused on mechanistic, ecophysiological insights into how conspecifics of different habitats (i.e. ecotypes) and latitude cope differently with energetics and flight performance after larval development under different thermal conditions. Our reciprocal transplant experiment under laboratory conditions with *P. aegeria* butterflies originating from two different habitat types at two different latitudes showed sex-specific effects of the influence of latitude and habitat type on metabolic rate and flight performance. Ecological context modulates physiology of organisms and affects their context-dependent performance through behaviour^32^. We will discuss i) the sexual dimorphism in metabolic rate relative to latitude and habitat and the significance of context-dependent plasticity; ii) flight performance in the flight mill relative to dispersal (and other movements); iii) the potential link with the pace-of-life concept; and iv) broader biogeographic and life history context of range dynamics within core areas of distribution ranges under climate change.

As predicted, patterns of SMR differed between males and females. The main effect of latitude on SMR independent of the thermal developmental conditions in males suggests a contribution of the genotype as we worked with F2 individuals. Northern or Belgian genotypes had higher SMR in males than southern or French genotypes, as predicted. However, females responded very differently as there was no effect of latitude on SMR, but a strong interaction effect between thermal conditions during larval development and the habitat type of origin. Females that originated from the ancestral woodland ecotype showed a canalized response for SMR relative to thermal treatment both for northern and southern latitudes. Females that originated from the agricultural landscape ecotype showed a much more plastic response, with higher SMR after the warm, southern developmental treatment compared to the cool, northern developmental treatment. This result supports the idea of female-limited differences in the degree of phenotypic plasticity of SMR between the ecotypes. This difference was independent of latitude, at least at the spatial scale it was tested here. The mechanistic underpinning of latitudinal variation in metabolic rates is still poorly understood, but recent work on orchid bees indicates a significant role for flight muscle membrane composition in explaining variation in FMR^33^.

Flight performance as quantified in a flight mill showed a sexual signature. The thermal treatment had no effect on the distance flown in males but southern males were more mobile than northern ones, whereas in females we found significant interaction effects between the thermal treatment and both latitude and habitat type, respectively. Flight distance was reduced if females developed under ‘transferred’ thermal conditions relative to their latitude of origin. So, southern genotypes that were forced to develop under cooler, northern conditions flew shorter distances in the flight mill than after development under warmer, southern conditions, and *vice versa*. The only exceptions were females of southern agricultural landscape origin; they did not reduce significantly their locomotor performance after cooler, northern developmental conditions as did the southern woodland ecotype. The general pattern is largely in line with local adaptation, i.e. constraints on flight performance after a thermal, larval development that deviated from the environment of origin. This was most pronounced in females of southern woodland landscape. This result is interesting for studying the effects of climate change related-movements that may occur also within the core of the range, with southern types moving poleward; like French butterflies joining, and eventually, taking over Belgian populations. One could also note that the southern agricultural females performed relatively poorly under their natural conditions compared to the other groups. Anyway, our results lead to the hypothesis that the probability, success rate and speed to colonize or invade northern areas or populations – also within the core of the range – is likely to differ between the ecotypes^cf.^^34^. In another study, flight endurance was found to increase with latitude in the butterfly *Pieris brassicae*^29^, whereas we found an opposite pattern in *P. aegeria* males. This shows the relevance of the total behavioural context as males of *P. brassicae* are always patrollers throughout their range, whereas in *P. aegeria* perching and patrolling coexisted but in different proportions relative to latitude and habitat type^31^.

In line with the dispersal-related predictions, FMR showed a clear relationship with latitude in females only. Northern females had higher FMR than southern females, but without any signature of their ecotype of origin. In males there was a significant negative effect of age, but that was a covariate and not a factor of interest in our experiments; age-effects of insect flight performance at the physiological level are known since long^35^. Our results for SMR and FMR gave different patterns^cf.^^36^ indicating that they cannot be interchanged as surrogates, opposite to results in several endotherms.

Particularly in vertebrate endotherms, several recent studies have interpreted latitudinal variation in resting metabolic rate within the context of the pace-of-life concept as an integrated mechanistic framework to understand variation in life styles relative to thermal conditions^37^. Tropical birds were shown to have lower pace of life characteristics compared to temperate-zone birds at the inter- and intraspecific level^38^,^39^. If we would assume a similar relationship between SMR and pace of life in butterflies, then latitudinal effects tend to go in the same direction, but at a much smaller spatial scale (*c*. 500 km) within the core of the distribution range. Such generalizations should be cautious at this stage; the universal validity of using metabolic rates as fundamental drivers of ecological and evolutionary processes has recently been questioned^40^ and the assumption of resting or standard metabolic rate as a proxy for energetic constraints may depend upon the general model of energy management that is applicable to the study organism^41^. Although much of this work was developed with endothermic vertebrates as study systems, the issue of energy management in ectotherms (including flying heliotherms like butterflies) and what that signifies for the assumption of the use of SMR as proxy for energetic constraints relative to other life history traits and fitness components needs further attention^13^. Flight mills have been used before^42^, but we combined this performance measure with respirometry as the mill was constructed in a climatic chamber^43^. Several studies on the evolutionary ecology of insect flight implicitly assumed flight performance to be unidimensional, but different flight types correspond to different performances (e.g., speed, maneuverability, endurance^27^). The performance in the flight mill corresponds to flight endurance, which is relevant for dispersal or other longer bouts of flight like in male patrolling behaviour to locate females. Hence, this device does not provide a universal proxy for flight performance. Earlier work has addressed maneuverability and acceleration capacity, which are more relevant to male territorial perching behaviour, rather than flight endurance per se^27^. FMR showed significant heritability that was context dependent in another butterfly species^44^ and was influenced by genotype-by-environment interactions with temperature as relevant environmental factor^36^. As butterflies were tethered, one has to be cautious with the absolute values of our estimates of flown distances, but our principal interest is a comparison between ecotypes and sexes.

Phenotypic change of a local population can be as important as environmental variability to understand population dynamics at the local, regional or range scale^45^. Behavioural and physiological traits of founders can have strong impact on population traits of new local populations^46^. Combining butterfly monitoring schemes^47^ with phenotypic and genetic surveys at a wider geographic scale would be an interesting approach to better grasp the eco-evolutionary dynamics of range dynamics, beyond core-edge comparisons and providing much needed insights into the dynamics, admixture and replacements of southern and northern genotypes/phenotypes within populations across the organism’s range.

In conclusion, our study provides experimental evidence for the sex-specific role of ecological context (i.e., habitat) to modulate intraspecific ecophysiology and flight performance in a context of range dynamics in an ectothermic insect under niche expansion. We demonstrated the relevance of studying functional phenotypes and performance at the core of the range in a context of climate-related range dynamics.

## Methods

### Species and study system

*P. aegeria* is a Palearctic butterfly; in NW-Europe it increased both in distribution and abundance over the last few decades^48^. Females lay eggs singly on different grasses. Gravid females (n = 44) were captured in nine populations: four populations in north Belgium (north; 51.0°N) and five in France (South; 46.3°N) along one longitude (4–5°E). The distance between the closest northern and southern populations was 522 km and they are located in different agroclimatic zones with warmer temperatures and increased solar radiation in the south compared to the north. At each latitudinal zone (Belgium or France) we collected females in at least two woodland and two agricultural landscape populations (Belgium: 2 woodland populations: Peertbos Herentals and Merodebos Herenthout; 2 agricultural landscape populations: Geel Ten Aert and Morkhoven – France: 2 woodland populations: Forêt de Villard and Bois Février; 3 agricultural landscape populations: Corgens, La Prairie and Laiz). Females were kept in small individual cages (30 cm^3^) and transported to a climate room; they had access to a 10% honey solution and a tuft of potted host grass (*Poa trivialis* reared under standardized conditions in the green house). Conditions in the climate room (Photoperiod: L:D 16 h:08 h; day temperature: 24.0 ± 0.8 °C and night temperature: 15.5 ± 0.5 °C; Philips HPI-T 400 W lamps were switched on at 7h00 Central European Time) induced direct larval development^49^.

The offspring (i.e., F1 generation) was reared on potted *P. trivialis* under the same light and thermal conditions (33 families in total; north-woodland: 8; north-agri: 9; south-woodland: 6; south-agri: 10 – average larval density per plant ± 1 SD = 6.4 ± 2.6; max = 8; N_plants_ = 79). Next, we created a F2 generation from which individuals were used for the experiments of this article (37 families in total; north-woodland: 8; north-agri: 9; south-woodland: 11; south-agri: 9). We did not cross between the different latitudes or between the ecotypes and excluded sib mating. At the day of oviposition, F2 eggs were transferred to a Petri dish. Four freshly hatched sibling larvae were placed on a potted tuft of *P. trivialis*. They were placed in one of two thermal treatments in two incubators (Sanyo MIR-554) following a split-brood design.

### Breeding experiment and thermal treatment

The first thermal treatment corresponded to a cool larval developmental regime as in the north (average day temperature: 17.1 ± 0.1 °C; average night temperature: 12.3 ± 0.6 °C) and the other to a warmer developmental regime as in the south (average day temperature: 22.1 ± 0.1 °C; average night temperature: 17.1 ± 0.5 °C). The applied values for the day temperatures were close to the long-term average ambient temperatures as recorded in nearby meteorological stations in summertime^22^. Larvae were fed *ad libitum* and the position of plants in the incubators was frequently changed. One-day old pupae were transferred to the climate room in individual plastic recipients.

### Standard metabolic rate

SMR is the lowest metabolic rate of an ectotherm while in rest, measured at a specified ambient temperature^11^. SMR was measured in 156 females and 151 males on the day after adult emergence (Sample sizes females/males per category: north-woodland-cool: 19/23; north-woodland-warm: 11/14; north-agri-cool: 16/16; north-agri-warm: 20/13; south-woodland-cool: 25/20; south-woodland-warm: 24/22; south-agri-cool: 24/26; south-agri-warm: 17/17). Before the experiment, butterflies were kept in individual cages (30 cm^3^), had free access to moist cotton wool and were weighted to the nearest 0.1 mg (Ohaus Balance). Next, the individual was placed in a 25-cl transparent plastic metabolic chamber connected to a stop-flow respirometer with a CO_2_ analyzer (Sable Systems Co) and with an internal thermoprobe (Physitemp probe type MT-29/1B connected to a Physitemp BAT-12 thermometer). Raw data from the system and the thermoprobe were processed by a UI-2 analogue/digital converter (Sable Systems) and stored using Sable Systems Expedata software. Ambient temperature (±SD) was 30.6 ± 0.7 °C (range: 28.7–33.8 °C). Note that the test temperature was chosen to reflect the likely body temperature of the butterflies when warmed by the sun and able to fly, which is considerably above the ambient temperature of the environment^12^,^50^. The individual was left in the chamber to acclimatize (25 min). The chamber was covered with a non-transparent cloth; as butterflies are heliotherms and sunlight triggers their active flight behaviour, they typically remain inactive under damped conditions, as was confirmed in our experiment. CO_2_-free dry air was pumped through the respirometer system. Next, the chamber was closed for 10 min and the CO_2_ emitted by the resting butterfly accumulated. The chamber was then flushed again (flow rate: 1981 ± 37 ml/min; Sable Systems subsample TR-SS3 pump) and the previously accumulated CO_2_ measured for 2 min (i.e., until reaching an almost null CO_2_-level). After the measurement of SMR, we measured simultaneously flight performance and FMR.

### Flight performance

Flight performance of 111 females and 95 males (Sample sizes females/males per category: north-woodland-cool: 14/16; north-woodland-warm: 10/6; north-agri-cool: 14/13; north-agri-warm: 12/7; south-woodland-cool: 21/16; south-woodland-warm: 16/19; south-agri-cool: 11/14; south-agri-warm: 13/4) was measured in a flight mill with a lightweight carbon rod arm (length: 290 mm; diameter: 1 mm) threaded through a stainless steel pivot (magnets) providing near-frictionless movement^43^. The number of revolutions was recorded by a Hall Effect sensor (Labview v 8.6 software) and recalculated as the distance flown. Each butterfly was gently attached to the flight mill arm, which was equipped with a counterbalance. The butterfly was first anaesthetized with CO_2_ (10 sec exposure) to remove carefully a dorsal zone of thoracic hairs and cuticular lipids with a fine cotton swab soaked with 90 °C ethanol. The bent tip of a needle was carefully glued on the pterothoracic segment (glue: Cyanocrylate ZAP PT09) and the upward pointing end of the needle fitted into a freely hinging holder at one side of the arm of the flight mill. Next, the 7-L chamber containing the flight mill, was closed (i.e., metabolic chamber connected to respirometer system). Ambient temperature ( ± SD) in the chamber was kept at 30.8 ± 0.6 °C (range: 29.1–33.6; within the voluntary flight activity range of body temperatures^12^).

### Flight metabolic rate

Once the butterfly was attached and the chamber closed (<5 min of manipulation), the mill was blocked with a magnet on the roof of the chamber. The chamber was kept under a light source (Philips HPL-R 400 W) and covered with a non-transparent cloth for *c*. 20 min (i.e. time needed to flush out CO_2_). Then we removed the cloth and closed the metabolic chamber to let CO_2_ accumulate during the performance test. Butterflies were allowed to fly during 10 min and stimulated to keep on flying by gently tapping on the chamber. The metabolic chamber was flushed again (flow rate: 1982 ± 31 ml/min) and the accumulated CO_2_ was measured for 9 min (i.e. until *c*. null CO_2_ level). Metabolic rates were calculated in ml CO_2_/h^12^. SMR was based on 12 min of CO_2_ emission at rest (10 min of accumulation and 2 min measurement) and FMR on 10 min of CO_2_ emission during flight.

### Data treatment and statistical analyses

Data were analyzed with Linear Mixed Models (following Box-Cox transformations if required; *P* > 0.05 for all Shapiro-Wilk tests on model residuals) in R software (v 3.0.1; nlme package). Analyses were done separately for males and females. Models tested the effects of latitude, habitat of origin (i.e., ecotype), developmental treatment and interaction effects. Age, temperature at testing and body mass were covariates. Full models of flight performance (i.e., distance flown) and FMR included total flight duration and testing time as covariates. The random factor population(family(paired larval rearing plant(larval rearing plant))) adjusted the analysis for the experimental design and pedigree (i.e., relatedness at the population and family level, common hatching date and rearing environment). Stepwise backward selection was applied selecting the minimal adequate model (keeping at least population(family) as random factor). We kept the model with the best fit based on the log-likelihood ratio test.

## Additional Information

**How to cite this article**: Van Dyck, H. and Holveck, M.-J. Ecotypic differentiation matters for latitudinal variation in energy metabolism and flight performance in a butterfly under climate change. *Sci. Rep.* **6**, 36941; doi: 10.1038/srep36941 (2016).

**Publisher’s note**: Springer Nature remains neutral with regard to jurisdictional claims in published maps and institutional affiliations.

## Supplementary Material

Supplementary Information

## Figures and Tables

**Table 1:** Summary of the linear mixed models (lme) for males testing for the fixed effects of latitude, habitat of origin, thermal treatment and body mass on SMR, flight performance (i.e. distance flown in the mill) and FMR.

Model terms	Estimate ± 1 SE	*F* (lme)	d.f.
SMR^a^			
Intercept	−7.693 ± 1.285	35.8***	1,117
Latitude (N > S)	0.077 ± 0.026	9.1*	1,10
Body mass (+)	0.028 ± 0.008	12.0***	1,117
Distance flown^a^			
Intercept	−2.778 ± 0.588	22.3***	1,64
Latitude (S > N)	−0.022 ± 0.010	5.0*	1,9
Total flight duration (+)	0.004 ± 0.001	39.5***	1,64
Body mass (+)	0.011 ± 0.003	14.7***	1,64
FMR^a^,^b^			
Intercept	−16.033 ± 10.413	2.4	1,59
Thermal treatment	0.589 ± 0.562	1.1	1,59
Latitude	0.213 ± 0.213	1.0	1,7
Habitat	25.491 ± 13.328	3.7	1,7
Age at testing (−)	−0.293 ± 0.093	10.0**	1,59
Total flight duration (+)	0.008 ± 0.001	37.6***	1,59
Body mass (+)	0.025 ± 0.006	20.3***	1,59
Thermal treatment × Latitude	−0.012 ± 0.011	1.0	1,59
Thermal treatment × Habitat	−1.370 ± 0.709	3.7 (*)	1,59
Latitude × Habitat	−0.509 ± 0.274	3.5	1,7
Thermal treatment × Latitude × Habitat	0.027 ± 0.015	3.0 (*)	1,59

(*)*P* < 0.07, *P < 0.05, **P < 0.01, ***P < 0.001. N, north; S, south; A, agriculture; W, woodland. There were no Type I errors as all significant variables in the minimal models were also significant in the full model without interactions.

^a^Population(family) as random factor.

^b^Following model selection after removing the marginally significant 3-way interaction, age at testing, total flight duration and body mass remain significant and there are no other significant factors.

**Table 2:** Summary of the linear mixed models (lme) for females testing for the fixed effects of latitude, habitat of origin, thermal treatment and body mass on SMR, flight performance (i.e. distance flown in the mill) and FMR.

Model terms	Estimate ± 1 SE	*F* (lme)	d.f.
SMR^a^			
Intercept	−25.479 ± 5.912	18.6**	1,118
Thermal treatment (+)	0.187 ± 0.058	10.4**	1,118
Habitat	3.119 ± 1.596	3.8	1,10
Age at testing (+)	0.490 ± 0.182	7.3**	1,118
Temperature at testing (+)	0.508 ± 0.184	7.7**	1,118
Body mass (+)	0.039 ± 0.009	11.3***	1,118
Thermal treatment × Habitat	−0.195 ± 0.082	5.7*	1,118
Distance flown^b^			
Intercept	−4.654 ± 2.086	5.0*	1,27
Thermal treatment	0.212 ± 0.107	3.9 (*)	1,22
Latitude (N > S)	0.097 ± 0.043	5.1*	1,9
Habitat (W > A)	−0.444 ± 0.207	4.6 (*)	1,9
Total flight duration (+)	0.001 ± 0.0002	16.8***	1,22
Body mass	0.002 ± 0.001	1.8	1,22
Thermal treatment × Latitude	−0.005 ± 0.002	4.5*	1,22
Thermal treatment × Habitat	0.024 ± 0.011	5.2*	1,22
FMR^a^			
Intercept	−2.256 ± 0.984	5.3*	1,77
Latitude (N > S)	0.039 ± 0.020	3.8 (*)	1,10
Body mass (+)	0.007 ± 0.004	3.4 (*)	1,77

(*)*P* < 0.07, *P < 0.05, **P < 0.01, ***P < 0.001. N, north; S, south; A, agriculture; W, woodland. There were no Type I errors as all significant variables in the minimal models were also significant in the full model without interactions.

^a^Population(family) as random factor.

^b^Population(family(paired larval rearing plant(larval rearing plant))) as random factor.
