# Supplementary material for: Ecotypic differentiation matters for latitudinal variation in energy metabolism and flight performance in a butterfly under climate change
Source: Sci Rep. 2016 Nov 15;6:36941. doi: 10.1038/srep36941 (PMC5109404; doi:10.1038/srep36941)
Supplement: Supplementary Information [file srep36941-s1.pdf]

SUPPLEMENTARY INFORMATION

Van Dyck, H. & Holveck, M.-J. Ecotypic differentiation matters for latitudinal variation in energy metabolism and flight performance in a butterfly under climate change. *Scientific Reports*

We added to the previously built linear mixed models (*see* Materials and methods) the standard metabolic rate SMR as predictor of flight metabolic rate FMR or of distance flown, and FMR as predictor of distance flown in a three-way interaction with latitude and habitat. In these analyses, we used body mass-corrected residuals for SMR, FMR and distance flown. SMR and FMR were not added together as predictors of distance flown to prevent model over-parameterization. The additional fixed effects retained in the minimal models as compared to previous minimal models presented in Table 1 are highlighted in bold in Table S2.

Table S1. Summary of the linear mixed models (lme) testing for the fixed effects of latitude, habitat of origin, and thermal treatment on the difference between FMR and SMR for each sex separately.

Model terms	Estimate ± 1 SE	*F* (lme)	d.f.
Males [a,b]			
Intercept	-19.469 ± 12.746	2.3	1,58
Thermal treatment	0.757 ± 0.688	1.2	1,58
Latitude	0.265 ± 0.261	1.0	1,7
Habitat	29.951 ± 16.367	3.3	1,7
Age at testing (-)	-0.325 ± 0.114	8.2**	1,58
Total flight duration (+)	0.010 ± 0.002	33.9***	1,58
Body mass (+)	0.023 ± 0.007	11.4**	
Thermal treatment x Latitude	-0.015 ± 0.015	1.1	1,58
Thermal treatment x Habitat	-1.629 ± 0.870	3.5(*)	1,58
Latitude x Habitat	-0.601 ± 0.336	3.2	1,7
Thermal treatment x Latitude x Habitat	0.033 ± 0.018	3.3(*)	1,58
Females [a]			
Intercept	-2.752 ± 1.140	5.8*	1,75
Latitude (N > S)	0.047 ± 0.023	4.0(*)	1,10
Body mass	0.005 ± 0.004	1.6	1,75

(*)$P < 0.07$, *$P < 0.05$, ** $P < 0.01$, ***$P < 0.001$. N, north; S, south. There were no Type I errors as all significant variables in the minimal models were also significant in the full model without interactions.

[a] Population(family) as random factor.

[b] Following model selection after removing the marginally significant 3-way interaction, age at testing, total flight duration and body mass remain significant and there are no other significant factors.

Table S2. Summary of the linear mixed models (lme) testing for the fixed effects of latitude, habitat of origin, and thermal treatment on each phenotypic trait for each sex separately (i.e. same models as in Table 1, but using residuals against body mass for each dependent variable), and including standard metabolic rate SMR (residuals against body mass) as predictor of flight metabolic rate FMR (residuals against body mass) or distance flown (residuals against body mass), and FMR as predictor of distance flown in a three-way interaction with latitude and habitat.

a) Males

Model terms	Estimate ± 1 SE	*F* (lme)	d.f.
Distance flown [a,b] with SMR [b] as predictor			
Intercept	-1.739 ± 0.295	34.7***	1,64
**SMR [b] (-)**	-0.074 ± 0.024	9.8**	1,64
Total flight duration (+)	0.003 ± 0.0005	34.4***	1,64
Distance flown [a,b] with FMR [b] as predictor			
Intercept	-0.040 ± 0.421	0.01	1,64
Latitude (S > N)	-0.020 ± 0.007	9.3*	1,9
**FMR [b] (+)**	0.203 ± 0.033	37.1***	1,64
Total flight duration (+)	0.002 ± 0.0005	12.7***	1,64
FMR [a,b,c] with SMR [b] as predictor			
Intercept	-12.906 ± 10.519	1.5	1,60
Thermal treatment	0.490 ± 0.568	0.7	1,60
Latitude	0.179 ± 0.215	0.7	1,7
Habitat	25.041 ± 13.466	3.5	1,7
Age at testing (-)	-0.259 ± 0.094	7.6**	1,60
Total flight duration (+)	0.007 ± 0.001	28.5***	1,60
Thermal treatment x Latitude	-0.009 ± 0.012	0.7	1,60
Thermal treatment x Habitat	-1.337 ± 0.717	3.5(*)	1,60
Latitude x Habitat	-0.499 ± 0.277	3.2	1,7
Thermal treatment x Latitude x Habitat	0.0266 ± 0.015	3.3(*)	1,60

b) Females

Model terms	Estimate ± 1 SE	*F* (lme)	d.f.
Distance flown [b,d] with SMR [b] as predictor			

Intercept	-6.358 ± 2.845	5.0*	1,26
Thermal treatment	0.270 ± 0.147	3.4	1,23
Latitude (N > S)	0.126 ± 0.058	4.7(*)	1,9
Habitat (W > A)	-0.677 ± 0.282	5.8*	1,9
Total flight duration (+)	0.001 ± 0.0002	20.7***	1,23
Thermal treatment x Latitude	-0.006 ± 0.003	4.0(*)	1,23
Thermal treatment x Habitat	0.037 ± 0.015	6.4*	1,23
Distance flown [b,d] with FMR [b] as predictor			
Intercept	-7.381 ± 2.294	10.3**	1,27
Thermal treatment	0.347 ± 0.119	8.6**	1,22
Latitude (N > S)	0.150 ± 0.047	10.2*	1,9
Habitat (W > A)	-0.579 ± 0.227	6.5*	1,9
**FMR [b] (+)**	0.235 ± 0.036	43.6***	1,22
Total flight duration (+)	0.0009 ± 0.0002	20.2***	1,22
Thermal treatment x Latitude	-0.008 ± 0.002	9.8**	1,22
Thermal treatment x Habitat	0.032 ± 0.012	7.3*	1,22
FMR [a,b] with SMR [b] as predictor			
Intercept	-1.862 ± 0.973	3.7(*)	1,77
Latitude (N > S)	0.038 ± 0.020	3.7(*)	1,10

(*)$P < 0.07$, *$P < 0.05$, ** $P < 0.01$, ***$P < 0.001$. N, north; S, south; A, agriculture; W, woodland.

There were no Type I errors as all significant variables in the minimal models were also significant in the full model without interactions. The terms in bold are the additional fixed effects retained in the minimal models as compared to previous minimal models presented in Table 1.

[a] Population(family) as random factor.

[b] Residuals of the linear regression of SMR, distance flown or FMR on body mass following Box-Cox transformation.

[c] Following model selection after removing the marginally significant 3-way interaction, age at testing and total flight duration remain significant and there are no other significant factors.

[d] Population(family(paired larval rearing plant(larval rearing plant))) as random factor.

Fig. S1. Two-by-two relationships of standard metabolic rate SMR, flight metabolic rate FMR and distance flown (all three as residuals against body mass) of males (a,c,e) and females (b,d,f) by latitude and ecotype of origin (north-woodland: squares, plain black line; north-agri: circles, dashed black line; south-woodland: triangles, plain grey line; south-agri: diamonds, dashed grey line).

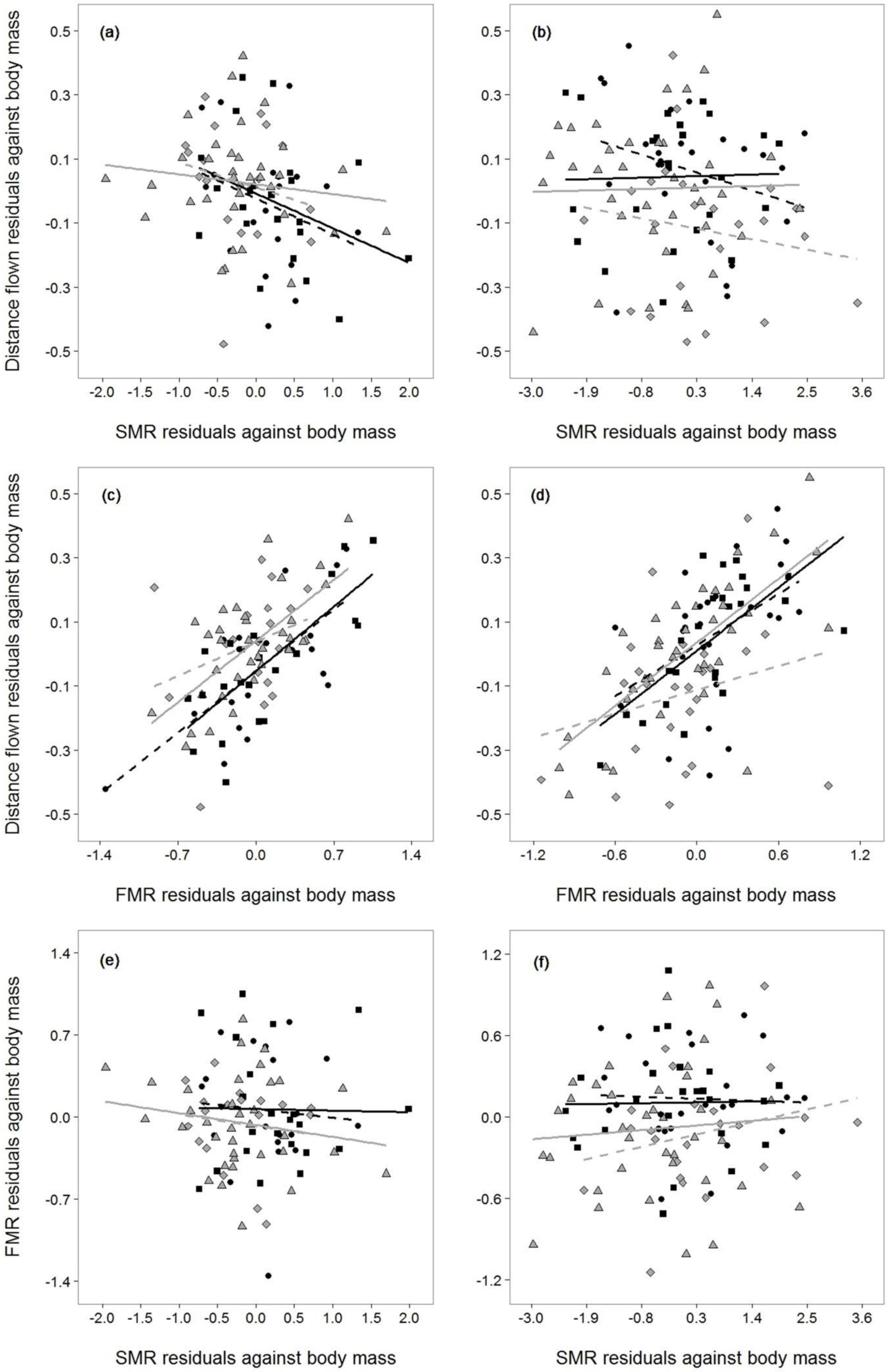
(a)
(b)
(c)
(d)
(e)
(f)
Distance flown residuals against body mass
FMR residuals against body mass
SMR residuals against body mass
FMR residuals against body mass
